# Epidemiology of sudden infant death syndrome in Mexico, 2005–2020

**DOI:** 10.3389/fped.2022.1001089

**Published:** 2022-12-08

**Authors:** Libny Martínez-Valdez, Vesta Richardson, Aurora Bautista-Márquez, Mauricio Hernández-Ávila

**Affiliations:** Dirección de Prestaciones Económicas y Sociales, El Instituto Mexicano del Seguro Social, Mexico City, Mexico

**Keywords:** epidemiology, sudden unexpected infant death (SUID), sudden infant death syndrome—SIDS, Mexico, children death

## Abstract

**Background:**

Sudden Infant Death Syndrome (SIDS) constitutes one of the main causes of mortality in children under one year of age in developed countries; it's frequency to varies geographically. In Mexico the real incidence of SIDS is not known.

**Methods:**

National databases of deaths in children under one year of age, from 2005 to 2020, were analyzed, due to Sudden Unexpected Infant Death (SUID) [SIDS (R95), accidental suffocation in a sleeping environment (W75), and other ill-defined and unspecified causes of mortality (R99), according to the International Classification of Diseases, tenth revision (ICD 10)]. Mortality rates per year of occurrence due to SUID and their subcategories were calculated. Simple frequencies of SIDS were obtained per year and month of occurrence, state of residence, age, place of death, and access to social security services.

**Results:**

In the study period 473,545 infant deaths occurred; 7,714 (1.62%) deaths were due to SUID; of these, 6,489 (84%) were due to SIDS, which is among the 10 leading causes of infant death in Mexico. The average mortality rate for SUID was 22.4/100,000 live births, for SIDS was 18.8/100,000 live births. Mortality rates within the states were variable, ranging from 2.4/100,000 to 105.1/100,000 live births. In 81% of SIDS records there was no autopsy; 38% of deaths due to SIDS occurred in infants under one month of age, up to 87% of deaths occurred in families without social security services or it was unknown, and 76.2% of deaths occurred at home. Deaths were more frequent during the last months of autumn and during winter.

**Conclusion:**

In Mexico there is an underregistry of SIDS as cause of death, along with other SUID categories. Health workers need to be trained to improve diagnosis and data registration, including the practice of autopsies; additionally, it is necessary to implement a public health campaign.

## Introduction

SIDS is defined as “the sudden unexpected death of an infant younger than one year of age, with onset of the fatal episode apparently occurring during sleep, that remains unexplained after a thorough investigation, including performance of a complete autopsy and review of the circumstances of death and the clinical history” (1–10).

SIDS must be considered a specific subcategory of Sudden Unexpected Infant Death (SUID) (11). In the last several years, the Centers for Disease Control in the US (CDC) suggested that SUID should be used as a broad term that encompasses all sudden infant deaths (12) whether explained or unexplained, which occur during the first year of life (2, 3, 13), including Sudden Infant Death Syndrome (SIDS), accidental suffocation in a sleeping environment (ASSB), and other deaths from unknown causes (14–16).

Therefore, for any SUID, when the cause of death after case investigation is not attributed to any explained cause such as suffocation, asphyxia, infection or metabolic diseases, the case must be classified as SIDS, which is an ultimate diagnosis reached by exclusion (15).

In developed countries, SIDS is the most common cause of death between 30 days and 12 months of life (4, 17) and represents 40%–50% of deaths in this age group (17).

Multiple studies have described the risk factors for SIDS, which are shown in Table 1.

**Table 1:** Risk factors for SIDS.

Risk Factors with genetic predisposition	Risk Factors with prenatal inﬂuence	Postnatal Risk Factors
• Ethnicity (Afro-American) (18)	• Smoking mother during pregnancy. Double risk (19, 20)	• Sleep prone (sleep on the stomach) or on side (21). Various epidemiological studies have identified the sleep prone position as the main risk factor for SIDS, with an OR ranging from 1.7 to 12.9
• Male gender (possibly linked to mutations on the X chromosome). 2:1 male predominance (17)	• Low socioeconomic status (17, 22)	• Age. Higher incidence from two to four months of age (18, 21)
• SIDS's brother victim. Risk of recurrence five times greater (17)	• Inappropriate prenatal control (17)	• Symptomatology of respiratory or gastrointestinal viral infection days before death (18)
• Genetic polymorphisms: Certain polymorphisms have been identified in genes of victims of SIDS, which interact with specific environmental factors (serotonin transport gene, genes encoding ionic channels of the heart and their relationship with arrhythmias, long QT syndrome, genes related to development of the autonomic nervous system, immune system dysfunctions) (17, 23–27)	• Abuse of heroin, cocaine and other drugs (28)	• Prematurity. Increase the risk four times (28, 29)
	• Multiparous mother with a pregnancy interval of less than one year (18)	• Low birth weight (28, 29)
	• Alcohol abuse during pregnancy (17)	• Anemia (18)
	• Maternal obesity (18)	• History of apnea (18)
	• Teenage mother (18)	• Exposure to tobacco smoke (17, 19, 20)
	• Complications during childbirth (maternal anemia, placental abruption) (18)	• Not feeding with breast milk (17, 28, 30)
		• Seasonality. The frequency doubles in the autumn and winter months, in contrast to the hot and dry months. (increase in exposure to viral infections, as well as to the babies’ dressing habits, for example, excessive shelter) (8, 18)
		• Excessive bedding, soft mattress and stuffed toys in the crib (20, 31)
		• Bed sharing with parents or siblings increases the risk, especially if accompanied by smoking, alcohol intake, drugs and/or sedative or drugs (28)
Own production		

Public health campaigns with simple and low-cost recommendations, such as supine position for sleeping, have been established since the 1980s worldwide and have shown to significantly reduce mortality. SIDS incidence has been significantly reduced after these campaigns (4–6, 13, 17, 20, 30, 32–34) in several developed and developing countries, as shown in Table 2.

**Table 2:** Mortality rates due SIDS (deaths/100,000 births) by year.

	Risk reduction campaign began	1987	1990	2000	2005	2011	2012	2013	2014	2019
Netherlands	**1987**	91	56	12	10	–	–	–	6	–
Norway	**1990**	–	170	44	30	–	–	–	–	–
Australia	**1991**	249	181	51	32	–	–	20	–	–
Germany	**1991**	164	142	63	43	–	–	22	–	–
England/Wales	**1991**	240	170	41	35	23	22	24	19	16
Sweden	**1992**	–	100	30	23	–	20	–	–	–
Canada	**1993**	106	81	37	24	19	–	–	–	–
France	**1994**	185	–	–	–	–	25	–	–	–
US	**1994**	137	130	62	54	48	42	40	39	33
Japan	**1998**	10	30	27	16	–	–	12	–	–
South Korea	**N/A**	–	–	–	56	–	–	20	–	–
Spain	**2000**	30	–	–	–	–	11	–	–	–
Argentina	**2003**	–	81	53	49	–	–	–	–	–
Italy	**2008**	11	–	–	–	–	3	–	–	–
Poland	**N/A**	26	–	–	–	–	13	–	–	–

N/A, not available. References: (6, 17, 20, 30, 33, 35, 36).

A study analyzed mortality rates trends from 2005 to 2015 due to SUID, SIDS, unknown/unattended/unspecified cause and accidental threats to breathing in 14 western European countries; the total rate of SUID was estimated at 34.9 per 100,000 live births, ranging from 12 to 76.4 among countries; Austria, Finland, France, Germany, Italy, the Netherlands, Norway, Spain and the United Kingdom reduced their mortality rates from this cause and mortality rates due to SIDS also decreased, however mortality rates due to unknown/unattended/unspecified causes increased (34). This also has been documented in the US, SUID rates declined from 154.6 per 100,000 live births in 1990 to 90.1 in 2019; SIDS rates also declined from 130.2 in 1990 to 33.3 in 2019 (16), although SIDS mortality rates declined, ASSB mortality rates increased; researchers have hypothesized that the continued decline may be due to changes in reporting or better diagnostic practices (37–39).

In Mexico, the real incidence of SIDS is not known; it is not considered a public health problem, there are very few specific preventive programs and no public health campaigns to reduce this cause of death, which may be related to the fact that there is under-registration of the number of cases or misdiagnosis (21). SIDS implies ruling out other possible causes of death, so there must be a precise diagnostic protocol (4) which in most cases is not practiced. In Mexico when a death occurs outside the hospital, the forensic service determines the causes of death and issues the certificate, the statistics area encodes and reports to the “civil registry”, which concentrates the information and notifies to the health jurisdiction, where experts review the certificates to request rectification or ratification as applicable, to the ones who emitted the certificate. The forensic, civil registry and health jurisdiction areas notify their State parties to validate or rectify the death certificate, later the process is repeated in the central offices at the national level with the same purpose, to finally publish the data.

Current guidelines recommend molecular autopsy in cases without a conclusive cause of death, especially in infants. In these situations, cardiac arrhythmia of genetic origin is suspected as the most plausible cause of death (23, 24, 27, 40). It is important to carefully study the clinical history of the victim and the circumstances of his death, because in case of negative autopsy, the pathologist can promptly collect fresh blood and samples of tissues for genetic testing (41).

Mexico's health system is composed of two sectors: public (90% of the population) and private (10% of the population). Health services can be offered as part of social security services by Institutions such as the Mexican Social Security Institute (IMSS for its name in Spanish), Institute of Social Security and Services for State Workers (ISSSTE for its name in Spanish), Petróleos Mexicanos (PEMEX), Ministry of National Defense (SEDENA for its name in Spanish), Ministry of Marine (SEMAR for its name in Spanish), which provide services to workers in the formal sector of the economy; and the Ministry of Health (including the extinct Seguro Popular), institution that provides medical services to the poorest population without access to social security services. In 2020, 73.4% of the population had access to public health services (42).

The aim of this study is to estimate the mortality rate due to SIDS in Mexico and its epidemiological characteristics, in order to understand the magnitude of SIDS as a public health problem and prioritize preventive interventions.

## Materials and methods

This is a retrospective population-based study which includes data related to infant deaths and births occurred in Mexico between 2005 and 2020. Births from 2008 to 2020 and all deaths were obtained from databases of the National General Office for Health Information (DGIS for its name in Spanish), of the Ministry of Health (43, 44). Births from 2005 to 2007 were obtained from databases of the National Institute of Statistics and Geography (INEGI for its name in Spanish) (45).

Outcome measures were the total number of deaths that occurred in infants younger than one year of age and the number of deaths due to SUID, including R95, Sudden infant death syndrome (before 2014); R950, Sudden infant death syndrome with mention of autopsy; R959, Sudden infant death syndrome without mention of autopsy; R99, Other ill-defined and unspecified causes of mortality; W75, Accidental suffocation and strangulation in bed (ASSB), including suffocation and strangulation due to bed linen, mother's body and pillow (occurred at house, residential institution, school, another place, place not specified); according to the International Classification of Diseases, tenth revision (ICD 10) version 2019.

Main causes of death in children under one year of age between 2005 and 2020 were identified, to understand the characteristics and factors associated with SIDS over time.

Simple frequencies of deaths and mortality rates due to SUID [SIDS, accidental suffocation in a sleeping environment (ASSB), and other ill-defined and unspecified causes of mortality] were calculated according to year of occurrence. Mortality rates were calculated using the number of deaths due to SUID as numerator and the number of live births as denominator.

Simple frequencies of deaths due to SIDS were obtained per year of occurrence, by age in months, sex, necropsy record, place where death occurred, state of residence and type of health system that provided care. Also, mortality rates per 100,000 live births were calculated by year at national level, and the average mortality rates through 2005–2020 were calculated for each of the thirty-two states of Mexico, using the number of deaths due to SIDS as numerator and the number of live births as denominator.

The number of deaths due to SIDS per month from 2005 to 2020 were plotted to identify if there was any temporal pattern in the occurrence of these deaths.

## Results

**Figure 1:**
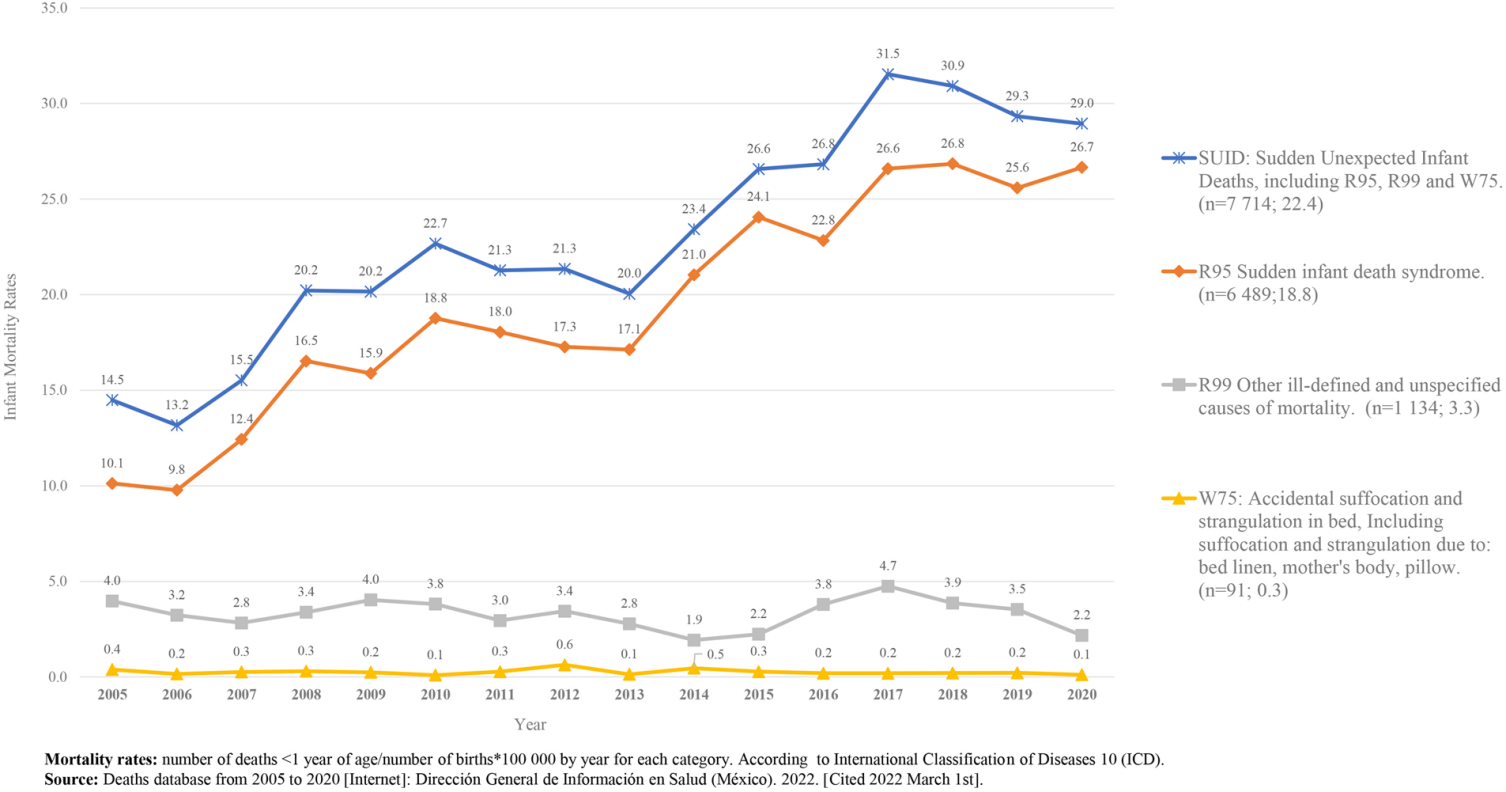
Infant mortality rates due to SUID. 2005–2020. México [Graph].

From 2005 to 2020, 473,545 infant deaths were recorded in Mexico; 7,714 (1.62%) deaths were due to SUID; of these, 6,489 (84%) were due to SIDS (an average of 405 deaths per year), 1,134 (14.7%) were due to other ill-defined and unspecified causes of mortality (R99), and 91 (1.17%) were due to ASSB (W75). The average mortality rates for the study period were: for SUID 22.4/100,000 live births, for SIDS 18.8/100,000 live births, for other ill-defined and unspecified causes of mortality 3.3/100,000 live births, and for accidental suffocation and strangulation in bed 0.3/100,000 live births (Figure 1).

**Figure 2:**
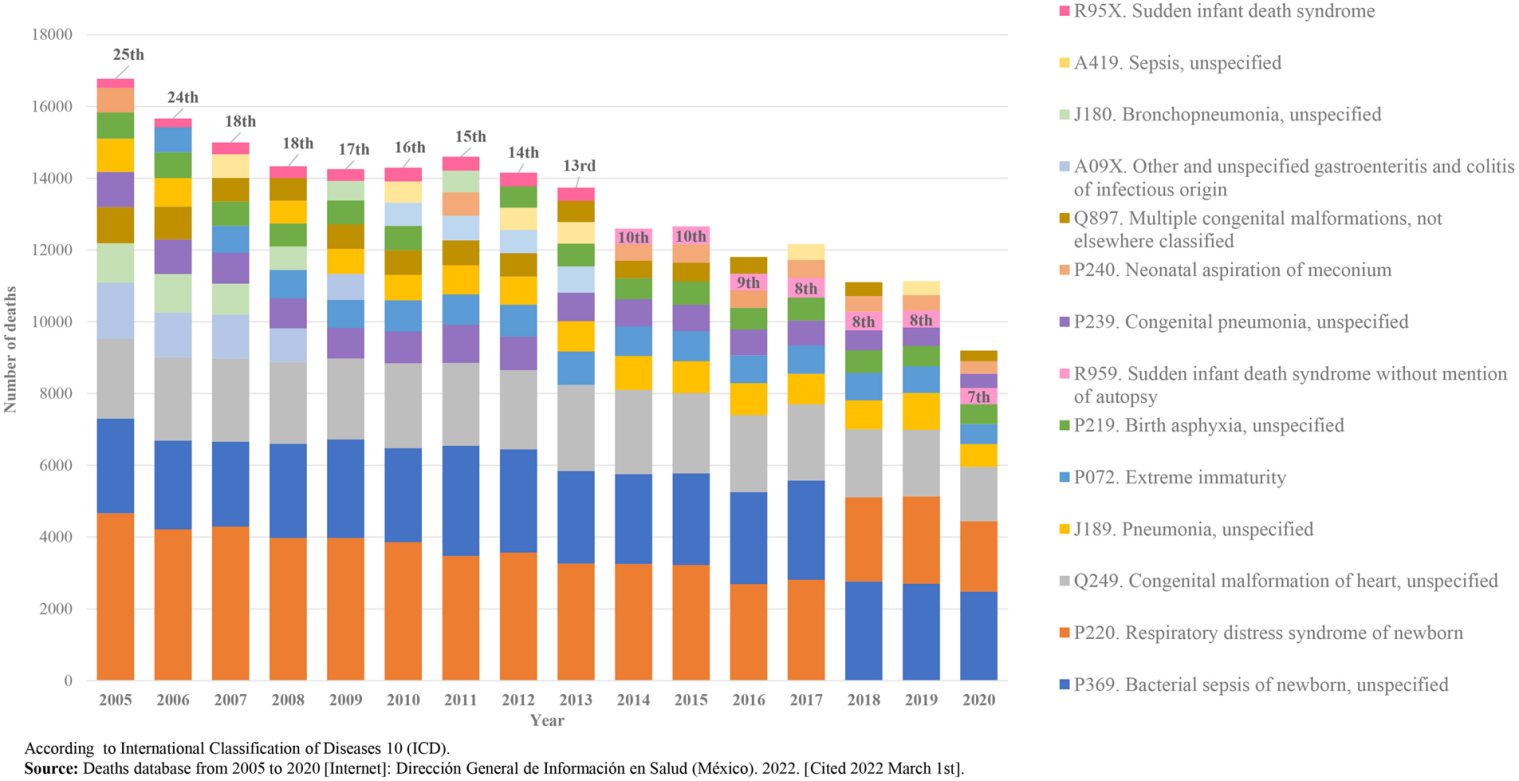
SIDS’ position as leading cause of death in children under one year of age, 2005–2020. Mexico. [Graph].

In Mexico, in 2005, SIDS was in the twenty fifth cause of death in children under one year of age and in 2020, it was the seventh cause of death (Figure 2).

We found that 5,255 (81%) SIDS cases did not have an autopsy recorded, in 883 (14%) this information was not specified and in 351 (5%) an autopsy was performed; of these cases, 212 (60%) were males, and 117 (33%) occurred in infants less than one month of age.

In relation to gender, 58% of deaths due to SIDS during the study period occurred in males.

**Figure 3:**
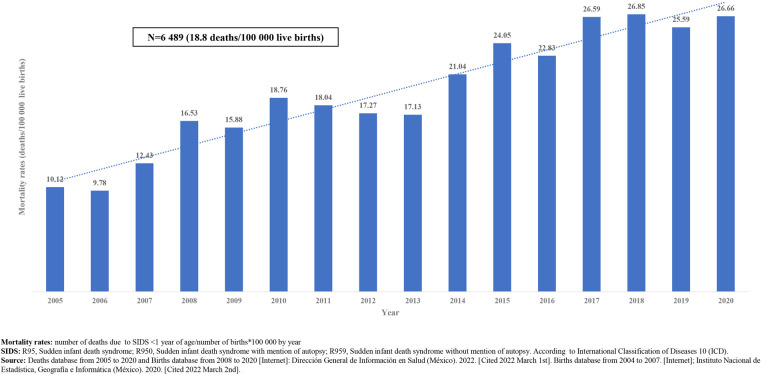
Mortality rates (deaths/100,000 live births) due to SIDS, 2005–2020. Mexico. [Graph].

The national average mortality rates due SIDS during the period shows that the trend goes up, with the highest national mortality rate, registered in 2018 (26.8 deaths/100,000 live births) Figure 3.

**Figure 4:**
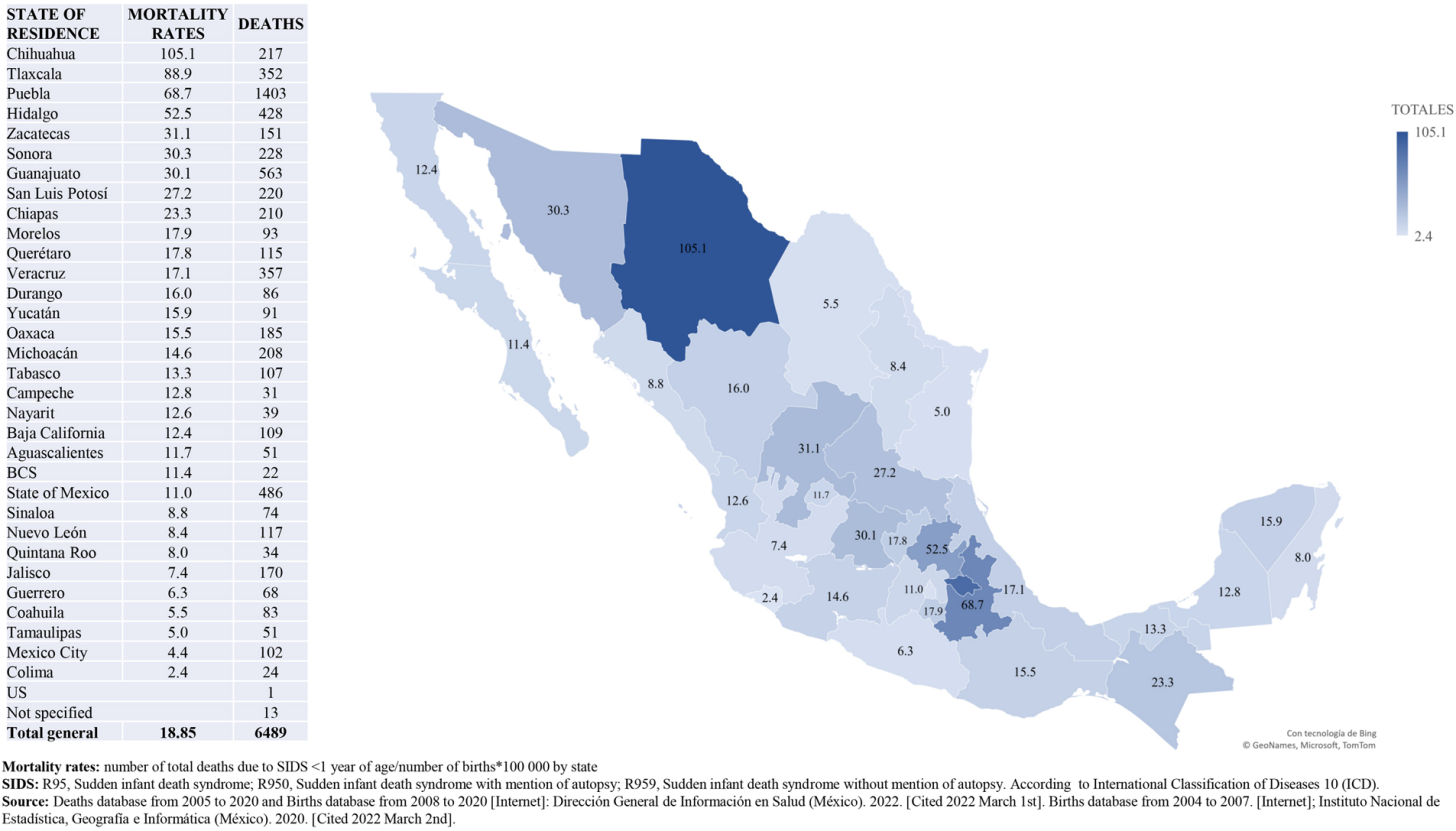
Mortality rates (deaths/100,000 live births) due to SIDS. State of residence, Mexico. 2005–2020 [Graph].

When analyzing data by state of residence, we found that there is high variability in the average mortality rate for the analyzed period, ranging from 2.4 deaths/100,000 live births in Colima (in the occidental coast of Mexico) to 105.1 deaths/100,000 live births in Chihuahua (in the north of Mexico). Additionally, we found that nine states have mortality rates higher than the national average mortality rate for the period, as shown in Figure 4. The trend of mortality rates due to SIDS in 29 states (91%) goes up and in three states (9%) the trend goes down (Chihuahua, México City and Querétaro).

**Figure 5:**
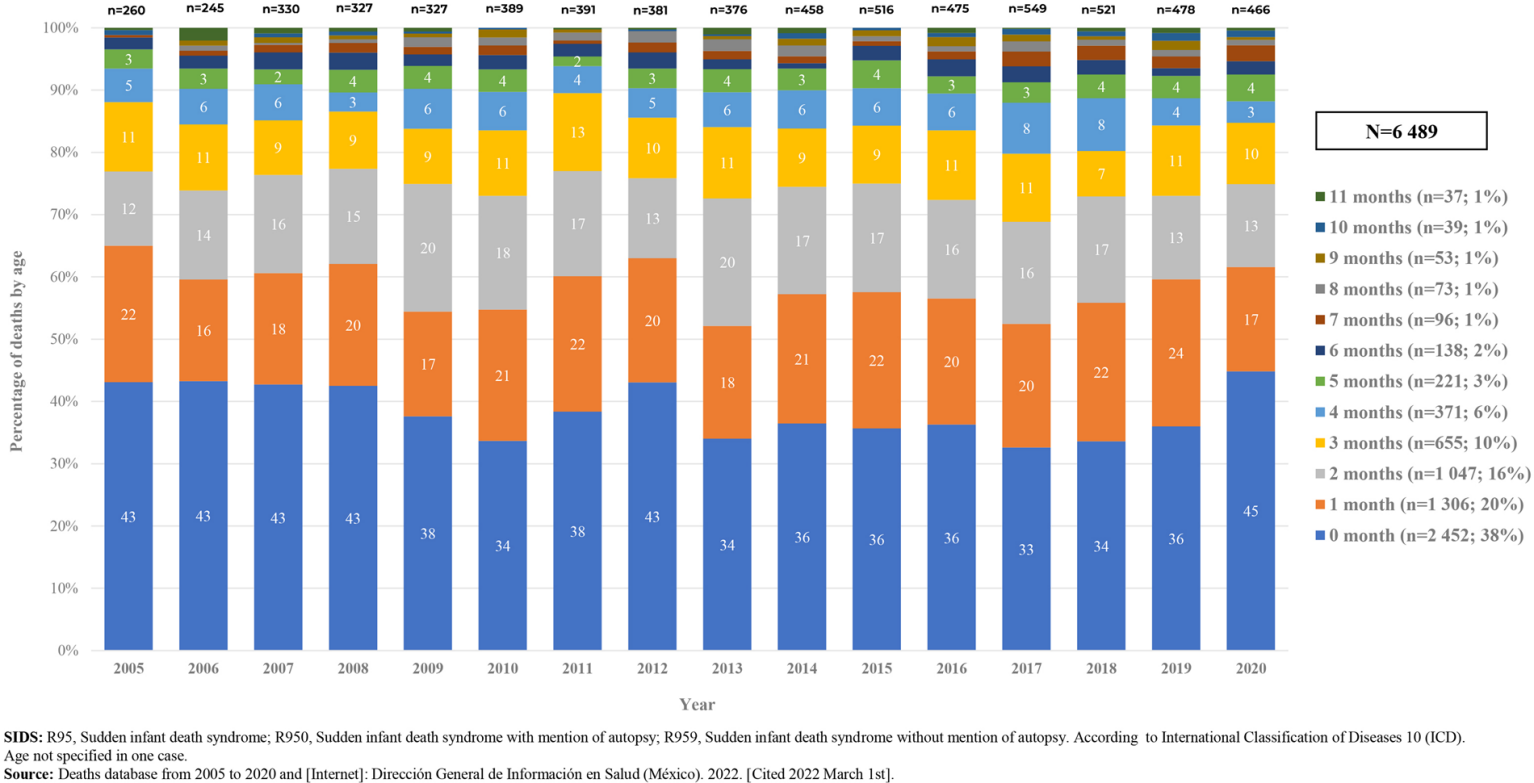
Deaths due to SIDS, by age, 2005–2020. Mexico. [Graph].

Of all deaths due to SIDS (6,489) during the study period, 2,452 (38%) occurred in infants under one month of age, 4,805 (74%) in infants less three months of age and 6,190 (95%) occurred during the first 6 months of life. Figure 5 shows distribution of deaths by age of occurrence pear year.

**Figure 6:**
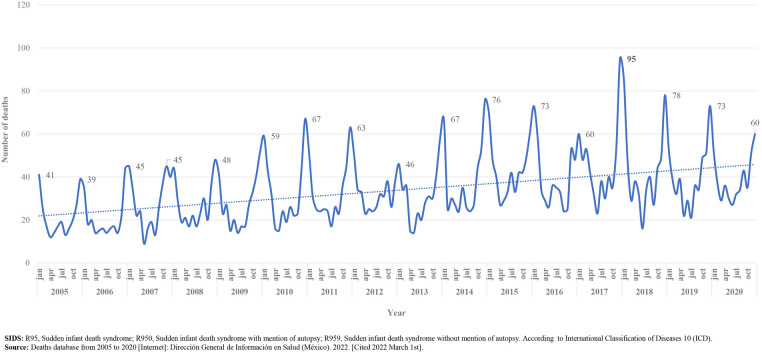
Deaths due to SIDS, monthly variation, 2005–2020. Mexico. [Graph].

As can be observed in Figure 6, deaths due to SIDS in Mexico, occurred throughout the year; however, most deaths happened from september/october to february/march; with a peak in december or january, with an average of 59–55 deaths in these months, respectively. The highest frequency was recorded in december 2017, with 95 deaths.

**Figure 7:**
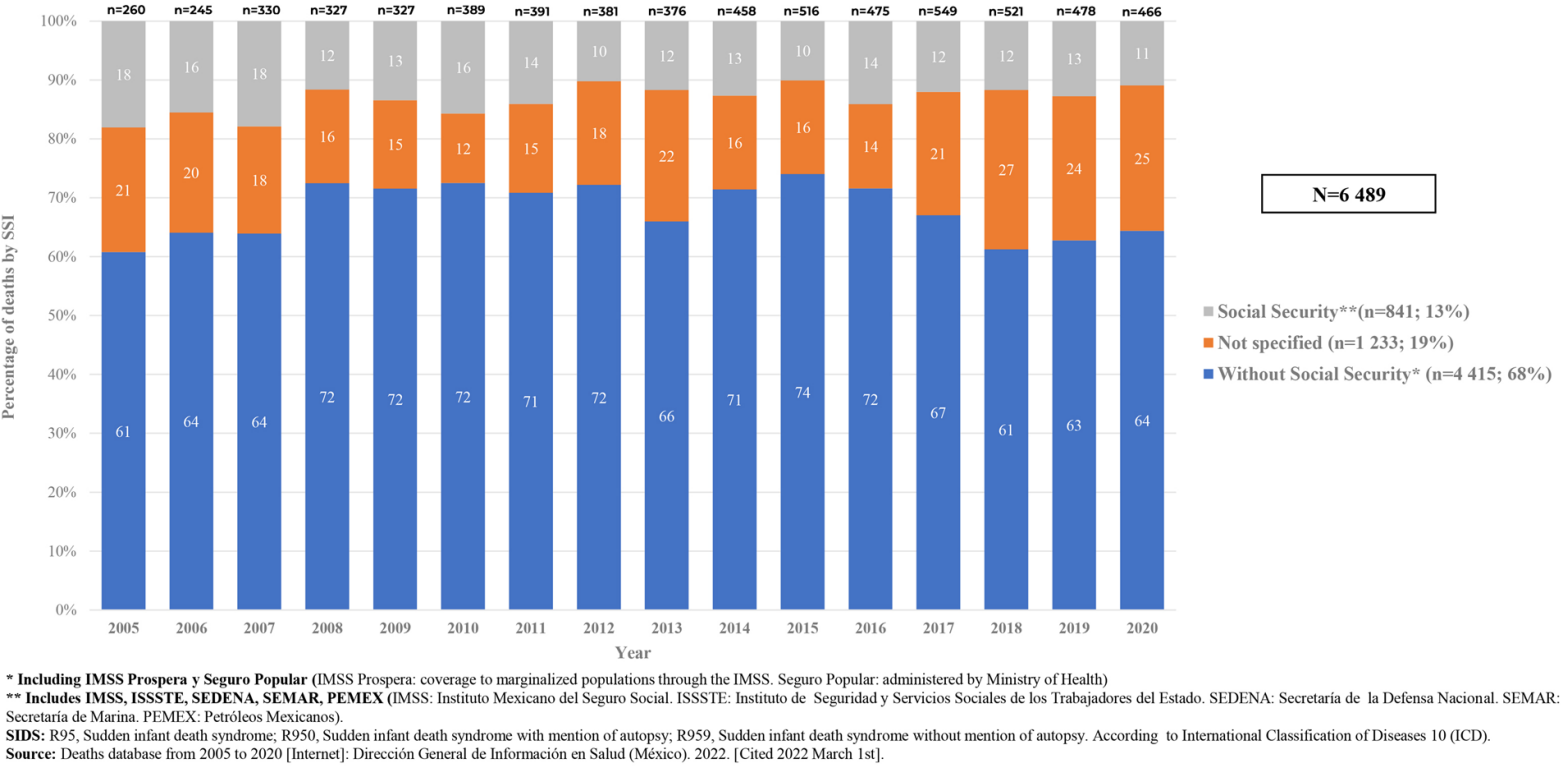
Deaths due to SIDS, by access to social security services, 2005–2020, Mexico. [Graph].

We also found that in the analyzed period (2005–2020), 4,948 (76.2%) of deaths due to SIDS occurred at home; 1,031(15.8%) occurred in a medical unit (public or private); 434 (6.6%) occurred in an unspecified place, and 76 (1.1%) occurred on public roads. 4,415 (68%) of deaths due to SIDS occurred in infants without social security services; 1,233 (19%) of deaths occurred in infants whose social security status was not specified or unknown; and 841 (13%) deaths occurred in social security Institutions (IMSS, ISSSTE, PEMEX, SEDENA, SEMAR) Figure 7.

## Discussion

In contrast to what has been seen in various countries where SIDS preventive campaigns have been implemented, in Mexico SIDS mortality rates have increased over time, and other diagnoses considered as part of SUID remain low and stable (Figure 1).

In Mexico, from 1998 to 2002, an average of 245 cases of SIDS/year (9 deaths/100,000 live births) were registered (21). From 2000 to 2011, the estimated average mortality rate was 13/100,000 live births (46). In our analysis, the estimated average mortality rate for the 2005 to 2020 period was 18/100,000 live births (Figure 3).

This increase in mortality rates may be due to excessive diagnosis without an exhaustive investigation of the underlying cause of death, including the analysis of the circumstances in which the death occurred, and the lack of an autopsy in the majority of cases. In addition, this may be due to inadequate training of forensic and medical staff and scarcity of human and economic resources (9, 10).

The fact that mortality rates due to SIDS in Mexico are much lower than those in the US and other developed countries (Table 2), suggests that there is still significant underregistry, underdiagnosis or misdiagnosis. Further research is needed to determine if race/ethnicity/genetic factors have a strong influence in the lower mortality rates, or if these are mainly related to prenatal and postnatal risk factors (Table 1).

In a European study, SUID was one of the five main infant causes of death, after those classified as “certain conditions originating in the perinatal period” and “congenital malformations or chromosomal abnormalities” (34). SIDS was the third leading cause of infant mortality in the US in 2020, after those classified as Congenital Anomalies and Short Gestation (47).

Another study compared the rates of SUID in several countries from 2002 to 2010 and found wide variability in how each country codes SUID, for example, the proportion of SUIDs coded as Sudden Infant Death Syndrome (R95) ranged from 32.6% in Japan to 72.5% in Germany (48). More recently, in European countries from 2005 to 2015, it ranges from 13.2% in Denmark to 67.7% in Belgium (34), while in our country SIDS represents 84.9% of the total SUID during the study period.

Since 2014, SIDS codification in Mexico includes the variable “with or without mention of autopsy”, and as shown in Figure 2, since 2014 SIDS has appeared within the ten main causes of death; this may be due to the substantial reduction in the number of deaths caused by gastrointestinal and respiratory disorders, infectious diseases, necrotizing enterocolitis and certain congenital malformations. More frequent than SIDS as causes of infant death in Mexico are congenital heart malformations, non-infectious respiratory disorders (respiratory distress syndrome of the newborn), birth asphyxia, newborn bacterial sepsis, unspecified pneumonia and disorders related to extreme immaturity.

We found high mortality rates due to SIDS in states of Mexico located at the north and centre of the country (Figure 4), states where during the winter season, the temperature is very cold. Additionally, as has been described in previous reports (3), we found that deaths have a specific temporal pattern: deaths accumulate during the last months of autumn and during the winter, with higher peaks in December and January (Figure 6). These two findings (geographic and seasonal distribution) are directly related to cold weather, season in which excessive clothing and/or bedding are used, and respiratory infections are more common, factors related with deaths due to ASSB or infection (38).

In our analysis, deaths due SIDS are more frequent in males, as described in other series (35).

It has been described that the most vulnerable period to die due to SIDS is within the first six months of age or before infants can roll over on their own (7). In our study, 95% of deaths classified as SIDS occurred in this period, as reported previously in countries such as Denmark, UK, Canada, US and Sweden (80%–90%) (3, 6, 21); nevertheless, we found that 38% of deaths due to SIDS occurred in infants under one month of age. In several reports' incidence of SIDS peaks between 2 and 4 months of age (5, 8, 21, 35). In our series, 58% of cases occurred during the first two months of age and as age progresses, the incidence decreased (Figure 5).

This analysis also shows that in Mexico, for the studied period, 76.2% of deaths due SIDS occurred at home and up to 87% occurred in families without social security services or where their social security status was not known (Figure 7). This fact is directly related to the socioeconomic status of families and formal jobs, and probably most of these deaths occurred among poor families (22, 49).

The loss of an apparently healthy baby is a catastrophic event for any family, disconcerting for a doctor and a challenge for the forensic expert or pathologist in charge of proving the cause of death (50), as well as for the person responsible for the categorization.

## Conclusion

SIDS in Mexico must be recognized as a public health problem that could be prevented with simple and non-expensive measures (19, 28–31, 51–54), as it has shifted to occupy one of the most common causes of infant death and mortality rates have doubled since 2005.

Our analysis suggests that in Mexico is an inaccurate classification of SIDS as cause of death, along with underregistry of other SUID categories, which makes it very difficult to estimate the real magnitude of these problems. Health workers need to be trained in SUID and SIDS definitions to improve diagnosis, coding and registry. Also the practice of autopsies according to specific protocols and consistency in application of an international classification system, is necessary.

Analysis of factors associated with deaths and the relationship with infant care practices in the general population are also needed for the development of more effective and appropriate public health campaigns, aimed to reach parents and caregivers within homes and childcare centers especially in children with known risk factors (55).

## Data Availability

Publicly accessible dataset were analyzed in this study. These can be found in Cubos dinámicos, Dirección General de Información en Salud. Available from: http://dgis.salud.gob.mx/contenidos/basesdedatos/BD_Cubos_gobmx.html
